# Urinary trace metals, maternal circulating angiogenic biomarkers, and preeclampsia: a single-contaminant and mixture-based approach

**DOI:** 10.1186/s12940-019-0503-5

**Published:** 2019-07-12

**Authors:** Paige A. Bommarito, Stephani S. Kim, John D. Meeker, Rebecca C. Fry, David E. Cantonwine, Thomas F. McElrath, Kelly K. Ferguson

**Affiliations:** 1 Environmental Science and Engineering, Gillings School of Global Public Health, University of North Carolina at Chapel Hill, 135 Dauer Drive, Chapel Hill, NC 27599 USA; 2 Epidemiology Branch, Division of Intramural Research, National Institute of Environmental Health Sciences, 111 T. W. Alexander Drive, Research Triangle Park, NC 27709 USA; 3 Department of Environmental Health Sciences, University of Michigan School of Public Health, Ann Arbor, MI 48109 USA; 4 Curriculum in Toxicology, School of Medicine, University of North Carolina at Chapel Hill, 104 Mason Farm Road, Chapel Hill, NC 27599 USA; 5 Division of Maternal-Fetal Medicine, Brigham and Women’s Hospital, Harvard Medical School, 75 Francis Street, Boston, MA 02115 USA

**Keywords:** Prenatal exposure, Metals, Metals mixtures, Angiogenic biomarkers, Preeclampsia

## Abstract

**Background:**

Exposures to toxic metals and deficiencies in essential metals disrupt placentation and may contribute to preeclampsia. However, effects of exposure to combinations of metals remain unknown.

**Objective:**

We investigated the relationship between urinary trace metals, circulating angiogenic biomarkers, and preeclampsia using the LIFECODES birth cohort.

**Methods:**

Urine samples collected during pregnancy were analyzed for 17 trace metals and plasma samples were analyzed for soluble fms-like tyrosine-1 (sFlt-1) and placental growth factor (PlGF). Cox proportional hazard models were used to estimate the hazard ratios (HR) of preeclampsia associated with urinary trace metals. Linear regression models were used to estimate the relationship between urinary trace metals and angiogenic biomarkers. Principal components analysis (PCA) was used to identify groups of metals and interactions between principal components (PCs) loaded by toxic and essential metals were examined.

**Results:**

In single-contaminant models, several toxic and essential metals were associated with lower PlGF and higher sFlt-1/PlGF ratio. Detection of urinary chromium was associated with preeclampsia: HR (95% Confidence Interval [CI]) = 3.48 (1.02, 11.8) and an IQR-increase in urinary selenium was associated with reduced risk of preeclampsia (HR: 0.28, 95% CI: 0.08, 0.94). Using PCA, 3 PCs were identified, characterized by essential metals (PC1), toxic metals (PC2), and seafood-associated metals (PC3). PC1 and PC2 were associated with lower PlGF levels, but not preeclampsia risk in the overall cohort.

**Conclusions:**

Trace urinary metals may be associated with adverse profiles of angiogenic biomarkers and preeclampsia.

**Electronic supplementary material:**

The online version of this article (10.1186/s12940-019-0503-5) contains supplementary material, which is available to authorized users.

## Background

Preeclampsia is a reproductive disorder that is diagnosed by de novo hypertension and proteinuria after 20 weeks gestation [1]. In the United States, the incidence of preeclampsia has increased over the past several decades and is estimated to affect approximately 5% of all pregnancies [2, 3]. Importantly, the underlying etiology of preeclampsia remains unknown. The origins of preeclampsia are hypothesized to lie within the placenta. Preeclamptic pregnancies are characterized by a placenta with shallow invasion into the maternal decidua and incomplete remodeling of maternal spiral arteries [3]. As a consequence, the preeclamptic placenta is poorly perfused and hypoxic, giving rise to the systemic maternal endothelial dysfunction that characterizes the disorder [3, 4].

Given that preeclampsia is characterized by impaired spiral artery remodeling, angiogenic biomarkers may serve as predictors of preeclampsia risk [5, 6]. These biomarkers include soluble fms-like tyrosine kinase-1 (sFlt-1) and placental growth factor (PlGF). Both sFlt-1 and PlGF are members of the vascular endothelial growth factor (VEGF) family and play important roles in adapting maternal spiral arteries during placentation. More specifically, higher levels of maternal circulating sFlt-1 indicate anti-angiogenic activity, while lower levels of circulating PlGF reflect reduced placental vascularization [7]. Both biomarkers have been shown to predict preeclampsia [8]. In addition, the sFlt-1/PlGF ratio (i.e., a ratio of anti-angiogenic to angiogenic activity) may more accurately predict preeclampsia than either biomarker alone [9].

Prenatal environmental metals exposure may contribute to preeclampsia risk. Previous studies have reported associations between toxic metals, such as cadmium (Cd) and lead (Pb), and an increased risk of preeclampsia [10–15]. Alternatively, higher exposure to essential metals, such as selenium (Se) and zinc (Zn), within a recommended range, have been routinely associated with a decreased risk of preeclampsia [16, 17]. Possible mechanisms for these associations include that toxic metals may impair trophoblast invasiveness [18–21], generate placental oxidative stress [22], or lead to maternal immunologic abnormalities [23], whereas essential metals have antioxidant properties that may reduce the effect of toxic exposures and promote normal placentation [24]. In addition, there are known toxicokinetic and toxicodynamic interactions between toxic and essential metals [25–27]. At an epidemiologic level, this has been demonstrated with respect to both placental functioning and preeclampsia, suggesting that essential metals may mitigate the relationship between toxic metals and preeclampsia [10, 28, 29].

While there is evidence for the role of metals in the development of preeclampsia, many of these studies come from populations with exceptionally high levels of toxic metals exposure [11, 12, 14]. Evidence is more limited in populations with lower exposures to toxic metals. In addition, while studies have examined relationships between individual trace metals and preeclampsia, very few studies have investigated metals in combination with one another [11]. In the present study, we performed an exploratory analysis of the relationship between 17 individual trace metals, maternal circulating levels of angiogenic biomarkers, and preeclampsia in the LIFECODES birth cohort. In addition, we investigated the impact of metals mixtures using principal components analysis (PCA) to examine the relationship between groups of urinary metals, preeclampsia, and levels of circulating angiogenic biomarkers. We selected PCA, rather than other statistical methods for analyzing chemical mixtures, as a mixtures-based approach as a method of dimension reduction and to examine groups of metals that may share important exposure sources or toxicities. Lastly, potential interactions between principal components characterized by toxic and essential metals were also investigated.

## Methods

### Subject recruitment

LIFECODES is an ongoing prospective birth cohort that began recruitment in 2006 at Brigham and Women’s Hospital (BWH) in Boston, MA. The study has few exclusion criteria. Specifically, women are eligible for enrollment into the LIFECODES cohort if they are at least 18 years of age, are seeking prenatal care before 15 weeks gestation, and intend on delivery at BWH [30]. At the first visit (median, 10 weeks gestation), women completed detailed questionnaires of demographic information and medical history and provided informed consent. Gestational age was defined according to the guidelines of the American College of Obstetricians and Gynecologists (ACOG), with the last menstrual period verified by first trimester ultrasound scanning [31]. Additional questionnaire data and urine samples are collected at three subsequent study visits (median, 18, 26, and 35 weeks gestation) and stored at − 80 °C until analysis [32]. All research was approved by the Institutional Review Board at BWH and was deemed exempt by the University of Michigan and the National Institute of Environmental Health Sciences (NIEHS).

The present analysis used subjects from a case-control study of preterm birth recruited from 2006 to 2008, which is nested in the parent LIFECODES cohort (Additional file 1: Figure S1). This case-control study included almost all cases of singleton preterm birth during this period and unmatched singleton controls were included in a 1:3 ratio [33]. The primary purpose of this case-control study was to examine the relationship between prenatal environmental exposures and preterm birth [34]. Participants in the case-control study were included in this analysis if they had urine samples from the 3rd study visit available for metals analysis (*n* = 390). Importantly, demographic characteristics in the subset of women with urine samples at the 3rd study visit is highly similar to both the larger case-control study of preterm birth [34] and the overall LIFECODES cohort [33]. To account for the disproportionate number of preterm births in our analysis, we applied inverse probability weighting based on preterm birth case status to all statistical analyses [35]. Thus, results are generalizable to what would have been observed in the parent LIFECODES birth cohort.

After delivery, preeclampsia diagnosis and diagnosis date were abstracted from medical records. Preeclampsia was defined according to ACOG guidelines between 2006 and 2008: elevated maternal blood pressure (> 140 mmHg systolic and/or > 90 mmHg diastolic) and proteinuria (> 300 mg/24 h or a protein/creatinine ratio > 0.20) after 20 weeks gestation [1]. All cases of preeclampsia were verified by two maternal-fetal medicine specialists and diagnosis date was used to determine the gestational age of preeclampsia onset. In the case of conflict, a third specialist reviewed the case. Among the 390 participants, 28 women developed preeclampsia. Among these 28 preeclampsia cases, one woman developed preeclampsia postpartum. For the present analysis, we excluded non-preeclamptics with gestational hypertension (*n* = 7) to provide a cleaner comparison of women who developed preeclampsia against women who did not develop any form of pregnancy-induced hypertension, resulting in a final sample size of 383.

### Urinary trace metals analysis

Urine samples from the 3rd study visit were sent to NSF International (Ann Arbor, MI, USA) for trace metals analysis, in collaboration with the Children’s Health Exposure Analysis Research (CHEAR) Program. Trace metals were analyzed using inductively coupled plasma-mass spectrometry (ICP-MS), with methods described in further detail elsewhere [32]. The metals analyzed included arsenic (As), barium (Ba), beryllium (Be), Cd, copper (Cu), chromium (Cr), mercury (Hg), manganese (Mn), molybdenum (Mo), nickel (Ni), Pb, Se, tin (Sn), thallium (Tl), uranium (U), tungsten (W), and Zn.

When metal concentrations were measured below the limit of detection (LOD) by ICP-MS, the machine-read values were used in analysis [32]. If the reported value was below zero or blank, the value was replaced with LOD/√2 [36, 37]. Metals with very low detection rates (< 30%) were represented as detect versus non-detect in all subsequent analyses.

### Plasma biomarkers of angiogenesis

Measurement of circulating maternal sFlt-1 and PlGF were measured in plasma samples collected at the 3rd study visit [5, 38]. Both sFlt-1 and PlGF were measured using ARCHITECT immunoassays (Abbot Laboratory, Abbott Park, IL, USA). Specifically, unbound PlGF concentrations from 1 to 1500 pg/mL were measured and total (unbound and bound) sFlt-1 concentrations from 0.10–150 ng/mL were measured. The ratio of sFlt-1 to PlGF was also calculated for analysis [9]. The combined intra- and interassay coefficients of variation were < 7% for both PlGF and sFlt-1 [5].

### Statistical analysis

First, differences in demographic and clinical characteristics based on preeclampsia status were examined using t-tests, chi-square tests, or Fisher’s exact tests, where appropriate. Second, we analyzed trace metal distributions by calculating median and interquartile ranges (IQR) by preeclampsia status.

Third, the relationship between urinary trace metals and the hazard ratio (HR) of preeclampsia were estimated using Cox proportional hazard models. Both unadjusted and adjusted Cox models were used to estimate the relationship between an IQR increase in urinary trace metals and the HR (95% Confidence Interval [CI]) of preeclampsia. The proportional hazards assumption was assessed by creating time-dependent variables for each predictor in the model. Tests of these time-dependent variables indicated that the proportional hazards assumption was satisfied. Trace urinary metals were log-transformed in order to improve model fit (i.e. lower AIC). Crude models were created with adjustment for gestational age at sample collection and, for metals that were modeled continuously, urinary specific gravity (SG). Potential confounders for adjusted analyses were identified using a directed acyclic graph (DAG) and included age (years), body mass index (BMI; kg/m^2^), maternal race (White/African American/Other), maternal education (< high school/technical college/ junior or some college/ > college), maternal health insurance status (private or HMO or self-pay/public), maternal tobacco use during pregnancy (yes/no), alcohol use during pregnancy (yes/no), parity (nulliparous/parous), previous preeclampsia diagnosis (yes/no), gestational diabetes (yes/no), use of assisted reproductive technology (ART; yes/no), chronic hypertension (yes/no), self-reported use of multivitamins (yes/no), calcium supplements (yes/no) and iron supplements (yes/no) during pregnancy, and infant sex (male/female) (Additional file 1: Figure S2). Factors were retained in adjusted analyses if their inclusion influenced effect estimates by > 10%. As a secondary analysis, we recreated the same statistical models excluding BMI as a covariate because of its potential role as a collider [39].

Fourth, the relationships between urinary trace metals and maternal angiogenic biomarkers were assessed using linear regression models. Trace metal species were log-transformed to improve model fit (i.e. lower AIC) and maternal angiogenic biomarkers were log-transformed to approximate a normal distribution. To ensure comparability between analyses, crude and adjusted models were constructed using the same confounders as the Cox models. Given that both the exposure and the outcome variables were log-transformed, beta estimates and standard errors from these regression models were converted to percent change (95% CI) associated with an IQR increase in trace metals concentration for interpretability.

Lastly, in addition to using single-contaminant models, urinary trace metals were investigated as a mixture using PCA. PCA was applied to the set of 13 urinary trace metals with > 30% detection to produce principal components (PCs). Urine metals measures were log-transformed and adjusted for SG prior to performing PCA to ensure normality and take the effect of hydration on the correlation of urinary metals into account. SG-correction was used to account for urine dilution using the following formula: Metal_SG_ = Metal[(1.015–1)/(SG-1)], where Metal_SG_ is the specific gravity-corrected metal concentration, Metal is the raw urinary trace metal concentration, 1.015 is the median specific gravity of all participants, and SG is the specific gravity level for the sample [40]. A combination of the “Eigenvalue-one” method and the Scree test was used to determine a meaningful set of components. Additionally, PCs were only retained if at least three trace metals loaded onto them with a factor loading > 0.40. Varimax rotation was used to maximize the variance in the factor pattern matrix [41]. While 13 urinary metals were initially included in PCA, Ba and Mo loaded onto multiple components, indicating that they were complex items [41]. Therefore, PCA was re-created excluding these metals. These PCA scores were then fit to [1] unadjusted and adjusted Cox proportional hazard models to estimate the relationship between a 1-unit change in PC score and the HR of preeclampsia, and [2] unadjusted and adjusted linear regression models to estimate the relationship between a 1-unit change in PC score and the percent change in circulating angiogenic biomarkers. Given that PCA revealed components loaded by toxic and essential metals, we examined interactions between groups of toxic and essential metals using nested interaction and main effects models. Significant interactions were identified using a likelihood ratio test (LRT). Interactions were considered significant if the LRT *p*-value < 0.10. For these models, the PC loaded by essential metals was dichotomized at the median (< median vs. > median) in order to report the association between toxic metals and preeclampsia among individuals with low vs. high levels of essential metals.

All analyses were performed using SAS version 9.4 (SAS Institute, Cary, NC, USA). Unless otherwise stated, significance was defined as *p*-value < 0.05.

## Results

### Subject demographics and urinary metals

Study sample demographics are summarized in Table 1. Briefly, the weighted study population had a median age of 32.7 years, a pre-pregnancy BMI of 23.9 kg/m^2^, was predominantly White (60.3%), and well-educated (86.5% postsecondary education). Women who developed preeclampsia were more likely to have a higher pre-pregnancy BMI (29.3 vs 23.8 kg/m^2^; *p* < 0.01), have been diagnosed with preeclampsia in a previous pregnancy (8.3% vs. 2.3%; *p* = 0.03), have used ART to become pregnant (24.3% vs. 8.3%; *p* = 0.02), have chronic hypertension (12.5% vs. 2.3%; *p* < 0.01), and be carrying a female fetus (77.2% vs. 42.8% male; *p* < 0.01). Women who developed preeclampsia were also more likely to report using calcium supplements during pregnancy (27.1% vs. 13.3%; *p* = 0.07) compared to women who did not. With respect to angiogenic biomarkers, circulating levels of PlGF were lower (275 vs. 456 pg/mL; *p* < 0.01) and the sFlt-1/PlGF ratio was higher (23.2 vs. 13.3; *p* = 0.04) among women who developed preeclampsia compared to those that did not. Interestingly, although previous analyses in the LIFECODES cohort have demonstrated differences in the trajectory of sFlt-1 levels between preeclamptics and non-preeclamptics throughout pregnancy [42], circulating sFlt-1 levels at the 3rd study visit did not differ by preeclampsia status.

**Table 1:** Weighted LIFECODES demographic characteristics overall and by preeclampsia status: crude N (weighted %) or weighted median (weighted IQR)

	Overall (*N* = 383)	Preeclamptic (*N* = 28)	Non-Preeclamptic (*N* = 355)	*P* ^*^
Maternal Age (years)	32.7 (29.1, 35.7)	33.0 (29.0, 35.1)	32.7 (29.1, 35.7)	0.62
Pre-pregnancy BMI (kg/m^2^)^a^	23.9 (21.4, 27.9)	29.8 (24.5, 36.7)	23.8 (21.3, 27.4)	< 0.01
Maternal Race				
White	231 (60.3%)	21 (75.0%)	210 (59.2%)	0.11
African American	57 (14.9%)	6 (19.4%)	51 (14.8%)	
Other	95 (24.8%)	1 (5.6%)	94 (26.1%)	
Maternal Education^a^				
< High School	53 (13.6%)	7 (21.5%)	46 (13.1%)	0.39
Technical College	57 (14.9%)	3 (9.7%)	54 (15.2%)	
Junior College or Some College	115 (31.3%)	11 (40.3%)	104 (30.7%)	
> College	148 (40.3%)	7 (28.5%)	141 (41.0%)	
Maternal Health Insurance^b^				
Private/HMO/Self-Pay	311 (82.7%)	25 (90.3%)	286 (82.2%)	0.31
Public	63 (17.3%)	3 (9.7%)	60 (17.8%)	
Maternal Smoking During Pregnancy^c^				
No	353 (93.9%)	24 (91.7%)	329 (94.0%)	0.56
Yes	25 (6.1%)	4 (8.3%)	21 (6.0%)	
Maternal Alcohol Use During Pregnancy^b^				
No	359 (95.2%)	28 (100.0%)	331 (94.9%)	
Yes	15 (4.8%)	0 (0.0%)	15 (5.1%)	
Parity				
Nulliparous	167 (43.8%)	12 (42.4%)	155 (43.9%)	0.88
Parous	216 (56.2%)	16 (57.6%)	200 (56.1%)	
Previous Preeclampsia Diagnosis				
No	370 (97.3%)	24 (91.7%)	346 (97.7%)	0.03
Yes	13 (2.7%)	4 (8.3%)	9 (2.3%)	
Gestational Diabetes				
No	355 (93.4%)	25 (90.3%)	330 (93.6%)	0.52
Yes	28 (6.6%)	3 (9.7%)	25 (6.4%)	
Use of ART				
No	349 (90.8%)	23 (75.7%)	326 (91.7%)	0.02
Yes	34 (9.2%)	5 (24.3%)	29 (8.3%)	
Chronic Hypertension				
No	368 (97.1%)	22 (87.6%)	346 (97.7%)	< 0.01
Yes	15 (2.9%)	6 (12.5%)	9 (2.3%)	
Use of Multivitamins During Pregnancy^c^				
No	106 (27.4%)	8 (30.6%)	98 (27.2%)	0.73
Yes	272 (72.6%)	20 (69.4%)	252 (72.8%)	
Use of Calcium Supplements During Pregnancy^c^				
No	324 (85.9%)	20 (72.9%)	304 (86.7%)	0.07
Yes	54 (14.1%)	8 (27.1%)	46 (13.3%)	
Use of Iron Supplement During Pregnancy^c^				
No	336 (88.7%)	24 (91.7%)	312 (88.5%)	0.53
Yes	42 (11.3%)	4 (8.3%)	38 (11.5%)	
Infant Sex				
Female	166 (44.7%)	17 (77.2%)	149 (42.8%)	< 0.01
Male	217 (55.3%)	11 (22.8%)	206 (57.2%)	
Maternal sFlt-1Expression^d^ (ng/mL)	5.79 (3.85, 9.12)	6.30 (4.15, 9.88)	5.72 (3.83, 9.06)	0.29
Maternal PLGF Expression^e^ (pg/mL)	448 (284, 645)	275 (421, 132)	456 (292, 651)	< 0.01
Maternal sFlt-1/PLGF Ratio^d^	13.8 (8.27, 22.9)	23.2 (12.0, 39.9)	13.3 (8.11, 22.3)	0.03

*Abbreviations*: *IQR* interquartile range, *BMI* body mass index, *ART* assisted reproductive technology, * sFlt-1* soluble fms-like tyrosine kinase-1, *PlGF* placental growth factor

*p-value for weighted t-test, Chi-square test, or Fisher Exact test, where appropriate

^a^ = 10 missing, ^b^ = 9 missing, ^c^ = 5 missing, ^d^ = 16 missing, ^e^ = 15 missing

Medians (IQRs) of SG-adjusted trace metals in 3rd study visit urine samples by preeclampsia status are shown in Table 2. Most trace metals had high detection rates. However, Be, Cr, U, and W were detected in < 30% of the samples. These metals were treated as detect vs. non-detect in all subsequent analyses. Overall, the distribution of metals in the urine was similar in women who developed preeclampsia and women who did not.

**Table 2:** Weighted distribution of specific gravity-adjusted trace metals from 3rd study visit urine samples (μg/L) by preeclampsia case status (*N* = 383)

Metals	LOD	N (%) < LOD	Preeclamptic Median (IQR) or N detected (weighted %)	Non-preeclamptic Median (IQR) or N detected (weighted %)
As	0.30	0 (0)	15.9 (6.05, 21.7)	17.9 (9.59, 32.6)
Ba	0.10	4 (1.03)	2.25 (0.93, 4.11)	1.93 (0.98, 3.34)
Cd	0.06	214 (55.9)	0.09 (0.06, 0.14)	0.08 (0.04, 0.14)
Cu	2.50	32 (8.21)	9.62 (8.77, 11.7)	8.96 (6.73, 12.1)
Hg	0.05	32 (8.21)	0.50 (0.24, 0.76)	0.51 (0.27, 0.97)
Mn	0.08	6 (1.54)	0.91 (0.53, 1.18)	0.73 (0.51, 1.13)
Mo	0.30	0 (0)	45.6 (36.2, 69.6)	51.3 (37.1, 68.8)
Ni	0.80	54 (13.8)	3.40 (1.86, 3.97)	2.84 (1.88, 3.97)
Pb	0.10	92 (23.6)	0.34 (0.16, 0.64)	0.35 (0.15, 0.62)
Se	5.00	3 (0.08)	36.3 (31.2, 46.0)	37.0 (29.6, 45.6)
Sn	0.10	24 (6.15)	0.47 (0.28, 0.98)	0.63 (0.35, 1.22)
Tl	0.02	61 (15.6)	0.13 (0.09, 0.16)	0.13 (0.08, 0.18)
Zn	2.00	0 (0)	294 (206, 398)	242 (146, 364)
Be^a^	0.04	356 (91.3)	3 (9.72)	31 (9.61)
Cr^a^	0.40	330 (84.6)	7 (21.5)	50 (13.2)
U^a^	0.01	342 (87.7)	4 (15.3)	43 (11.3)
W^a^	0.20	309 (79.2)	4 (15.3)	73 (19.5)

*Abbreviations*: *LOD* limit of detection, *IQR* interquartile range, *As* arsenic, *Ba* barium, *Cd cadmium*, *Cu* copper, *Hg* mercury, *Mn* manganese, *Mo* molybdenum, *Ni* nickel, *Pb* lead, *Se* selenium, *Sn* tin, *Tl* thallium, *Zn* zinc, *Be* beryllium, *Cr* chromium, *U* uranium, *W* tungsten

^a^Denotes metals with > 70% of samples below the limit of detection

### Urinary trace metals and preeclampsia risk

After adjusting for smoking during pregnancy, maternal race, maternal educational attainment, insurance status, infant sex, ART use, self-reported calcium supplementation, and pre-pregnancy BMI, the detection of Cr in urine was positively associated with preeclampsia risk (HR: 3.48, 95% CI: 1.02, 11.8) (Table 3). This association was similar in models excluding BMI (see Additional file 1: Table S1). However, it should be noted that Cr was only detected in 15% (*N* = 57) of all participants and 22% (*N* = 7) of the women who developed preeclampsia and this association is based on a limited number of observations. In addition, an IQR-increase in urinary Se was associated with a reduction in the risk of preeclampsia (HR: 0.28, 95% CI: 0.08, 0.94).

**Table 3:** Adjusted^a^ association between urinary metals and the HR (95% CI) of preeclampsia

	Adjusted^a^ HR (95% CI)	*p*
Single Contaminant Models		
As	0.73 (0.46, 1.16)	0.18
Ba	1.05 (0.62, 1.79)	0.85
Cd	0.94 (0.54, 1.64)	0.83
Cu	0.71 (0.23, 2.24)	0.56
Hg	0.90 (0.63, 1.28)	0.55
Mn	1.26 (0.75, 2.12)	0.39
Mo	0.47 (0.21, 1.04)	0.06
Ni	0.89 (0.50, 1.59)	0.69
Pb	0.97 (0.67, 1.40)	0.86
Se	0.28 (0.08, 0.94)	0.04
Sn	0.82 (0.48, 1.38)	0.45
Tl	0.80 (0.47, 1.37)	0.42
Zn	0.94 (0.44, 2.02)	0.88
Be^b^	1.46 (0.32, 6.74)	0.63
Cr^b^	3.48 (1.02, 11.8)	0.05
U^b^	0.99 (0.23, 4.21)	0.99
W^b^	1.77 (0.49, 6.37)	0.38
Principal Components Analysis Models		
PC1: Cu, Se, and Zn	0.89 (0.35, 2.29)	0.81
PC2: Cd, Mn, and Pb	1.63 (0.74, 3.61)	0.22
PC3: As, Hg, and Sn	0.75 (0.39, 1.46)	0.40

*Abbreviations*: *HR* hazard ratio, *CI* confidence interval, *PC* principal component, *As* arsenic, *Ba* barium, *Cd* cadmium, *Cu* copper, *Hg* mercury, *Mn* manganese, *Mo* molybdenum, *Ni* nickel, *Pb* lead, *Se* selenium, *Sn* tin, *Tl* thallium, *Zn* zinc, *Be* beryllium, *Cr* chromium, *U* uranium, *W* tungsten

^a^Adjusted for specific gravity (for continuously measured metals in single-contaminant models), smoking during pregnancy, race, educational attainment, insurance status, infant sex, ART, calcium supplementation, pre-pregnancy BMI, and gestational age at study visit

^b^Denotes metals with > 70% of samples below the limit of detection

### Urinary trace metals and circulating maternal angiogenic biomarkers

After adjusting for potential confounders, urinary Cu was associated with higher circulating sFlt-1 levels (% Change: 11.5, 95% CI: 0.18, 24.1) (Table 4). No other statistically significant associations were observed between urinary metals and sFlt-1 levels. Several metals were associated with lower levels of circulating PlGF. Namely, an IQR-increase in urinary Cd (% Change: -6.99, 95% CI: − 13.1, − 0.47), urinary Pb (% Change: -7.20, 95% CI: − 11.8, − 2.33), and detection of urinary Cr (% Change: -24.5, 95% CI: − 38.2, − 7.77) was associated with lower levels of circulating PlGF. In addition, urinary Cu and Se were also associated with lower circulating PlGF levels. Urinary Cu was also associated with a 23.7% (95% CI: 6.44, 43.8) higher sFlt-1/PlGF ratio (Table 4). Results from crude models and models excluding BMI remained highly similar (see Additional file 1: Table S2).

**Table 4:** Adjusted^a^ relationship between urinary trace metals and the percent change (95% CI) in circulating maternal angiogenic biomarkers

	sFlt-1		PlGF		sFlt-1/PlGF Ratio	
	% Change (95% CI)	*p*	% Change (95% CI)	*p*	% Change (95% CI)	*p*
Single Contaminant Models						
As	−0.88 (−7.07, 5.73)	0.79	0.05 (− 6.47, 7.03)	1.0	−1.96 (− 10.5, 7.39)	0.67
Ba	−0.95 (−7.24, 5.78)	0.78	−2.77 (− 9.23, 4.15)	0.42	2.06 (− 6.99, 12.0)	0.67
Cd	−0.45 (−6.72, 6.24)	0.89	−6.99 (− 13.1, − 0.47)	0.04	6.64 (− 2.71, 16.9)	0.17
Cu	11.5 (0.18, 24.1)	0.05	−10.6 (− 20.1, − 0.001)	0.05	23.7 (6.44, 43.8)	< 0.01
Hg	4.99 (−0.95, 11.3)	0.10	−1.65 (−7.48, 4.56)	0.59	6.17 (−2.22, 15.3)	0.15
Mn	1.45 (−5.89, 9.36)	0.71	−1.44 (−8.89, 6.62)	0.72	1.88 (−8.37, 13.3)	0.73
Mo	0.69 (−9.49, 12.0)	0.90	4.99 (−6.09, 17.4)	0.39	−5.72 (−18.9, 9.60)	0.44
Ni	2.27 (−6.46, 11.8)	0.62	−0.51 (−9.39, 9.24)	0.92	4.22 (− 8.12, 18.2)	0.52
Pb	−2.34 (−7.03, 2.58)	0.35	−7.20 (−11.8, − 2.33)	< 0.01	4.80 (− 2.24, 12.4)	0.19
Se	−4.07 (− 19.5, 14.3)	0.64	− 18.2 (−31.8, − 1.80)	0.03	15.9 (− 9.49, 48.3)	0.24
Sn	5.15 (− 1.17, 11.9)	0.11	4.98 (− 1.61, 12.0)	0.14	0.22 (− 8.20, 9.42)	0.96
Tl	−4.52 (−11.1, 2.56)	0.20	−4.35 (− 11.3, 3.08)	0.24	−0.30 (− 9.89, 10.3)	0.95
Zn	−2.67 (−11.22, 6.7)	0.56	−5.17 (− 13.9, 4.41)	0.28	3.45 (−9.16, 17.8)	0.61
Be^b^	−6.72 (−25.2, 16.3)	0.54	15.3 (−8.42, 45.2)	0.23	−17.5 (−39.5, 12.6)	0.23
Cr^b^	−9.09 (−25.4, 10.8)	0.34	−24.5 (−38.2, − 7.77)	< 0.01	5.25 (− 20.4, 39.1)	0.72
U^b^	−17.7 (− 33.3, 1.48)	0.07	− 9.16 (− 27.1, 13.1)	0.39	−11.1 (− 33.9, 19.5)	0.44
W^b^	1.09 (−14.0, 18.9)	0.90	−1.62 (− 16.9, 16.5)	0.85	4.77 (− 16.6, 31.7)	0.69
Principal Components Analysis						
PC1: Cu, Se, and Zn	−3.85 (−13.7, 7.07)	0.47	−13.5 (−22.6, − 3.41)	0.01	11.6 (− 3.97, 29.8)	0.15
PC2: Cd, Mn, and Pb	2.23 (−7.74, 13.3)	0.67	−11.9 (−20.7, − 2.12)	0.02	14.9 (−0.49, 32.8)	0.06
PC3: As, Hg, and Sn	4.59 (−5.71, 16.0)	0.40	3.43 (−7.03, 15.1)	0.54	0.56 (−13.0, 16.2)	0.94

*Abbreviations*: *sFlt-1* soluble fms-like tyrosine, *PlGF* placental growth factor, *HR* hazard ratio, *CI* confidence interval, *PC* principal component, *As* arsenic, *Ba* barium, *Cd* cadmium *Cu* copper, *Hg* mercury, *Mn* manganese, *Mo* molybdenum, *Ni* nickel, *Pb* lead, *Se* selenium, *Sn* tin, *Tl* thallium, *Zn* zinc, *Be* beryllium, *Cr* chromium, *U* uranium, *W* tungsten

^a^Adjusted for specific gravity (for continuously measured metals in single-contaminant models), smoking during pregnancy, race, educational attainment, insurance status, infant sex, ART, calcium supplementation, pre-pregnancy BMI, and gestational age at study visit

^b^Denotes metals with > 70% of samples below the limit of detection

### Trace metals mixtures, preeclampsia risk, and Angiogenic biomarkers

Using PCA, we identified three primary PCs, which explained 46.0% of the variance in urinary metals. PC1 was characterized by higher loading of essential metals (Cu, Se, and Zn); PC2 was characterized by higher loading of toxic metals (As, Mn, and Pb); and PC3 was characterized by higher loading of seafood-associated metals (As, Hg, and Sn) [43–45]. These factor loading patterns have been previously observed in our examination of urinary trace metals and preterm birth in this study population [32]. Factor loadings and communalities for urinary metals are displayed in (see Additional file 1: Table S3).

**Fig. 1:**
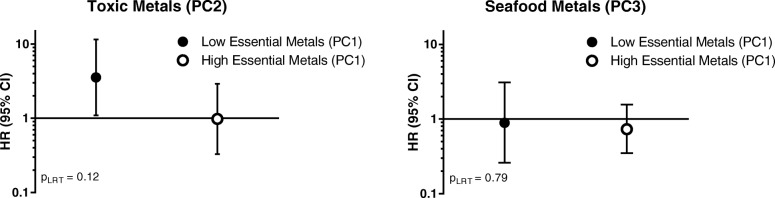
Adjusted^a^ HR (95% CI) of preeclampsia with PCs loaded by toxic metals (PC2) and seafood-associated metals (PC3) within strata of above vs. below median levels of essential metals (PC1) ^a^Adjusted for smoking during pregnancy, race, educational attainment, insurance status, infant sex, ART, calcium supplementation, pre-pregnancy BMI, and gestational age at study visit. Abbreviations: HR, hazard ratio; CI, confidence interval, PC, principal component, p_LRT_, likelihood ratio test *p*-value

With respect to preeclampsia, neither PC1 (Cu, Se, and Zn) (HR: 0.89, 95% CI: 0.35, 2.29), PC2 (Cd, Mn, and Pb) (HR: 1.63, 95% CI: 0.74, 3.61), nor PC3 (As, Hg, and Sn) (HR: 0.75, 95% CI: 0.39, 1.46) were statistically significantly associated with preeclampsia (Table 3). Interactions between PCs loaded by toxic/seafood-associated metals (i.e. PC2 and PC3) and PCs loaded by essential metals (PC1) were further investigated (Fig. 1). While there was no statistically significant interaction between PC1 (Cu, Se, and Zn) and PC2 (Cd, Mn, and Pb) (p_LRT_ = 0.12), the association between PC2 (Cd, Mn, and Pb) and preeclampsia (HR: 3.54, 95% CI: 1.09, 11.5) was significant among individuals with low levels of PC1 (Cu, Se, and Zn). On the other hand, this association was null among individuals with higher levels of PC1(Cu, Se, and Zn).

After adjusting for potential confounders, PC1 (Cu, Se, and Zn) (% Change: -13.5, 95% CI: − 22.6, − 3.41) and PC2 (Cd, Mn, and Pb) (% Change: -11.9, 95% CI: -20.7, -2.12) were associated with lower circulating PlGF levels (Table 4). The associations between PCs and angiogenic biomarkers were similar in both crude and adjusted models with BMI excluded (see Additional file 1: Table S2).

## Discussion

In this study, the relationships between 17 trace urinary metals, preeclampsia risk, and levels of maternal circulating angiogenic biomarkers were investigated using both a single-contaminant and mixtures-based approach. In our single-contaminant approach, we observed an increased risk of preeclampsia associated with the detection of Cr in the urine and a reduced risk of preeclampsia associated with an IQR-increase in urinary Se. Urinary Cd, along with other toxic and essential metals, was also associated with reductions in circulating PlGF levels and urinary Cu was associated with higher sFlt-1 levels and a higher sFlt-1/PlGF ratio, suggesting an association with impaired placentation and preeclampsia risk. In our mixtures approach, we used PCA to further investigate how combinations of urinary metals were associated with the risk of preeclampsia and circulating angiogenic biomarker levels. Individual PCs were not associated with preeclampsia risk, but both PC1 (Cu, Se, and Zn) and PC2 (Cd, Mn, and Pb) were associated with lower levels of circulating PlGF. In addition, while we did not observe a statistically significant interaction between PC1 (Cu, Se, and Zn) and PC2 (Cd, Mn, and Pb), the association between PC2 (Cd, Mn, and Pb) and preeclampsia was significant among individuals with low levels of PC1 (Cu, Se, and Zn).

In single-contaminant models, detection of Cr in the urine was associated with an increased risk of preeclampsia and a reduction in circulating PlGF levels, suggesting that Cr is associated with a reduction in placentation and placental function. While the effect estimates were relatively imprecise, other studies have also noted higher levels of total urinary or hair Cr in preeclampsia cases compared to normotensive controls [11, 15]. Urinary Cr levels in this cohort were low, with most samples being below the LOD, but the primary source of exposure to Cr in the general population is from food, particularly from meat and fish products [46]. Recent toxicologic studies have also demonstrated that hexavalent Cr exposure leads to increased expression of oxidative stress markers and apoptotic signaling in trophoblast cells and mouse placenta [47–49], providing further molecular-level evidence for a potential link between Cr exposure and preeclampsia. Despite the consistency of association between urinary Cr, angiogenic biomarkers, and preeclampsia, we did not feel that it was appropriate to apply mediation analysis methodologies to this cohort given the small number of samples with Cr above the LOD, the relatively high degree of imprecision in the estimates, and the lack of clear connection between Cr exposure and preeclampsia in current epidemiologic and toxicologic literature. Nevertheless, we feel that these results warrant further research.

Although we did not find associations between other toxic metals and the risk of preeclampsia, we identified associations between urinary Cd and Pb and lower circulating PlGF levels. These results may provide additional mechanistic evidence for previous studies that have reported associations between prenatal Cd and Pb exposure and preeclampsia [10, 13, 50]. Both Cd and Pb are ubiquitous environmental exposures. Both may be found in contaminated air, soil, or water. Cd levels tend to be higher in certain foods, such as rice and grains, and both Cd and Pb can be found in drinking water systems or housing, particularly in areas with old or failing infrastructure [51–53]. Similarly, the PC loaded by toxic metals (PC2: Cd, Pb, and Mn) was not associated with preeclampsia risk, but was associated with lower circulating PlGF levels.

With respect to essential metals, we observed a reduction, albeit imprecise, in the risk of preeclampsia associated with an increase in urinary Se. This observation is consistent with the literature, which suggests that essential metals, including Se, may reduce the risk of preeclampsia [16, 17]. In contrast, urinary Cu and Se were associated with lower circulating PlGF levels. Urinary Cu was also associated with higher sFlt-1 and the sFlt-1/PlGF ratio. Likewise, in our mixtures-based approach, the PC for essential metals (PC1: Cu, Se, and Zn) was associated with lower circulating PlGF levels. These findings may suggest that, instead of having a beneficial effect, levels of essential metals are associated with impaired placental angiogenesis and placentation. This is consistent with previously observed associations between urinary Cu and the risk of preterm birth, specifically placentally-mediated preterm birth, in this study population [32]. In addition, recent meta-analyses have reported consistent observations that higher plasma or serum Cu is associated with preeclampsia risk [54, 55]. While Cu is an integral component of the antioxidant system, modest increases in Cu may lead to the production of reactive oxygen species and oxidative stress, which has been linked to the development of preeclampsia [9, 56]. Alternatively, it has been suggested that higher levels of essential metals, including Cu, may be the result of preeclampsia, where women developing preeclampsia may increase the uptake of essential metals in attempt to buffer the higher levels oxidative stress associated with the disease [56].

In addition to PC1 (Cu, Se, and Zn) and PC2 (Cd, Mn, and Pb), we identified a third PC that was characterized by loading from seafood-associated metals (PC3: As, Hg, and Sn). Notably, while inorganic As is considered a toxic metal, non-toxic organic forms of As, such as arsenobetaine, are widely found in seafood [57]. In populations with frequent seafood consumption, including those within the United States, much of the total urinary As may be organic arsenicals from such foods [58–60]. In addition, although seafood-associated metals (PC3: As, Hg, and Sn) grouped independently of other toxic metals (PC2: Cd, Mn, and Pb), they should not be considered non-toxic. For instance, pregnant women are advised against consuming certain types of seafood on the basis that it may contain high levels of methylmercury [61]. Despite this, we did not identify associations between PC3 (As, Hg, and Sn) and either preeclampsia or angiogenic biomarkers. Disentangling the harmful effects of these trace metals from the beneficial effects of seafood consumption (i.e. fatty acids) is complex and beyond the scope of this analysis.

Given known toxicologic interactions between toxic and essential metals, we investigated statistical interactions between toxic and essential metals with respect to both preeclampsia risk and levels of circulating angiogenic biomarkers. While there was no significant interaction between PC1 (Cu, Se, and Zn) and PC2 (Cd, Mn, and Pb), the association between PC2 (Cd, Mn, and Pb) and preeclampsia was significant among individuals with lower levels of PC1 (Cu, Se, and Zn). Previously, significant interactions between placental Cd and Se levels were reported in a case-control study of preeclampsia [10]. At a molecular level, these findings are supported by evidence of toxic and essential metals interactions within the placenta. For example, antagonistic interactions between maternal toenail Cd and Se levels have been noted with respect to placental apoptotic gene expression [29]. Taken together, these results may suggest that individuals with low levels of essential metals may be more susceptible to the effects of toxic exposures on placental development and/or functioning.

This study has several strengths. First, the LIFECODES birth cohort has a prospective study design. Urine samples were collected at the 3rd study visit and no patients had developed preeclampsia at the time of sample collection, reducing the likelihood that these results were influenced by reverse causality. Nevertheless, it is possible that preeclampsia was subclinical at the time of sample collection (median, 26 weeks gestation) because the origins of preeclampsia may lie in the early stages of pregnancy. Second, this study is among the first to employ a mixtures approach in the context of preeclampsia. This is of critical importance given that populations are exposed to complex mixtures of toxicants, rather than single-contaminants, and represents a major public health research objective [62].

Despite these strengths, this study also has several limitations. First, this study had a small number of preeclampsia cases. Thus, it was not possible to examine the relationship between urinary metals and individual subtypes of preeclampsia (i.e., early- vs. late-onset). There is evidence that these subtypes may have different underlying etiologies [63]. It should also be noted that the sampling design of the case-control study that this analysis is based in may have resulted in an oversampling of early-onset preeclampsia. However, this oversampling should be partially accounted for by the inverse weighting approach used to account for selection into the study on preterm birth status. In addition, the limited sample size precluded an investigation of different dose-response shapes for trace metals and preeclampsia. Some, such as Mn, may feature non-monotonic dose-response curves that are not well captured by linear modeling. Second, with respect to exposure assessment, biomarkers of exposure were only available as urinary measures and at a single timepoint. Exposure to some metals may be better captured using other matrices, such as blood biomarkers for Se, Mn, Zn or Pb exposure [64–67]. Moreover, multiple metals measures taken throughout pregnancy would have allowed for a better characterization of metals exposure throughout pregnancy. In particular, metals measures taken earlier during pregnancy may better capture exposure at the time of early preeclampsia development. Third, we used PCA as a mixtures-based approach to examine urinary trace metals and preeclampsia. Like all statistical methods for examining chemical mixtures, PCA has limitations [68]. Unsupervised PCA reduces dimensionality by selecting linear combinations of variables without considering how they relate to the outcome of interest. Thus, while it identifies chemicals with similar exposure patterns, it may not have selected the most relevant groups of trace metals for preeclampsia or angiogenic biomarkers. Moreover, because exposure patterns vary across populations, the PCs identified in this study may not be generalizable to other studies. In addition, by assuming linear combinations of variables within a PC, it assumes no interactions between metals within each cluster. Lastly, the generalizability of this study may be limited given that participants were recruited from BWH, a tertiary care center, which receives higher-risk pregnancies than other hospitals. However, this population does exhibit similar concentrations of urinary trace metals to those observed in the general United States population [32].

## Conclusions

This study investigated 17 urinary metals, individually and in mixtures, in association with circulating maternal angiogenic biomarkers and preeclampsia. This study is among the first to examine the relationship between prenatal trace metals exposure and maternal angiogenic biomarkers, as well as the relationship between metals mixtures and preeclampsia. The results from both our single-contaminant and mixtures-based approach suggests that both toxic and essential urinary metals are associated with adverse maternal profiles of angiogenic biomarkers. In addition, there may be important interactions between toxic and essential metals in the context of preeclampsia.

## Additional file

Additional file 1: **Figure S1.** Flow-through diagram displaying study design. **Figure S2.** Simplified directed acyclic graph (DAG) used to identify potential confounders and/or colliders. **Table S1.** Unadjusted and adjusted relationship between urinary metals and the HR (95% CI) of preeclampsia. **Table S2.** Unadjusted and adjusted relationship between urinary trace metals and the percent change (95% CI) in circulating maternal angiogenic biomarkers. **Table S3.** Standardized and rotated loading factors and communalities for each variable within each principal component. (DOCX 208 kb)

## Data Availability

The datasets generated and analyzed during the current study are not publically available due to the sensitive nature of human biological and environmental exposure data, but are available from the corresponding author upon reasonable request.

## Abbreviations

ACOG: American College of Obstetricians and Gynecologists
ART: Assisted reproductive technology
As: Arsenic
Ba: Barium
Be: Beryllium
BMI: Body mass index
BWH: Brigham and Women’s Hospital
Cd: Cadmium
CHEAR: Children’s Health Exposure Analysis Research
CI: Confidence interval
Cr: Chromium
Cu: Copper
DAG: Directed acyclic graph
Hg: Mercury
HR: Hazard ratio
ICP-MS: Inductively coupled plasma-mass spectrometry
IQR: Interquartile range
LOD: Limit of detection
LRT: Likelihood ratio test
Mn: Manganese
Mo: Molybdenum
Ni: Nickel
NIEHS: National Institue of Environmental Health Sciences
Pb: Lead
PC: Principal components
PCA: Principal components analysis
PlGF: Placental growth factor
Se: Selenium
sFlt-1: soluble fms-like tyrosine kinase-1
SG: Specific gravity
Sn: Tin
Tl: Thallium
U: Uranium
VEGF: Vascular endothelial growth factor
W: Tungsten
Zn: Zinc
