# Supplementary material for: Urinary trace metals, maternal circulating angiogenic biomarkers, and preeclampsia: a single-contaminant and mixture-based approach
Source: Environ Health. 2019 Jul 12;18:63. doi: 10.1186/s12940-019-0503-5 (PMC6624941; doi:10.1186/s12940-019-0503-5)
Supplement: Supplementary file 1 — Figure S1. Flow-through diagram displaying study design. Figure S2. Simplified directed acyclic graph (DAG) used to identify potential confounders and/or colliders. Table S1. Unadjusted and adjusted relationship between urinary metals and the HR (95% CI) of preeclampsia. Table S2. Unadjusted and adjusted relationship between urinary trace metals and the percent change (95% CI) in circulating maternal angiogenic biomarkers. Table S3. Standardized and rotated loading factors and communalities for each variable within each principal component. (DOCX 208 kb) [file 12940_2019_503_MOESM1_ESM.docx]

| **Supplemental Material** |
| --- |
| **Urinary Trace Metals, Maternal Circulating Angiogenic Biomarkers, and Preeclampsia: A Single-Contaminant and Mixture-Based Approach** |
|  |
|  |
| Paige A. Bommarito^1^, Stephani S. Kim^2^, John D. Meeker^3^, Rebecca C. Fry^1,4^, David E. Cantonwine^5^, Thomas F. McElrath^5^, Kelly K. Ferguson^2*^ |
|  |
|  |
|  |
| ^1^Environmental Science and Engineering, Gillings School of Global Public Health, University of North Carolina at Chapel Hill, Chapel Hill, NC, USA. |
| ^2^Epidemiology Branch, National Institute of Environmental Health Sciences, Research Triangle Park, NC, USA |
| ^3^Department of Environmental Health Sciences, University of Michigan School of Public Health, Ann Arbor, MI, USA |
| ^4^Curriculum in Toxicology, School of Medicine, University of North Carolina at Chapel Hill, Chapel Hill, NC, USA |
| ^5^Division of Maternal-Fetal Medicine, Brigham and Women’s Hospital, Harvard Medical School, Boston, MA, USA |
|  |
|  |
|  |
|  |
| *Corresponding Author. |
| [Email: kelly.ferguson2@nih.gov](mailto:kelly.ferguson2@nih.gov) |
| Ph: 984-287-3700 |
| Mail: 111 TW Alexander Drive. P.O. Box 12233 Mail Drop A3-05 Durham, NC 27709 |

**
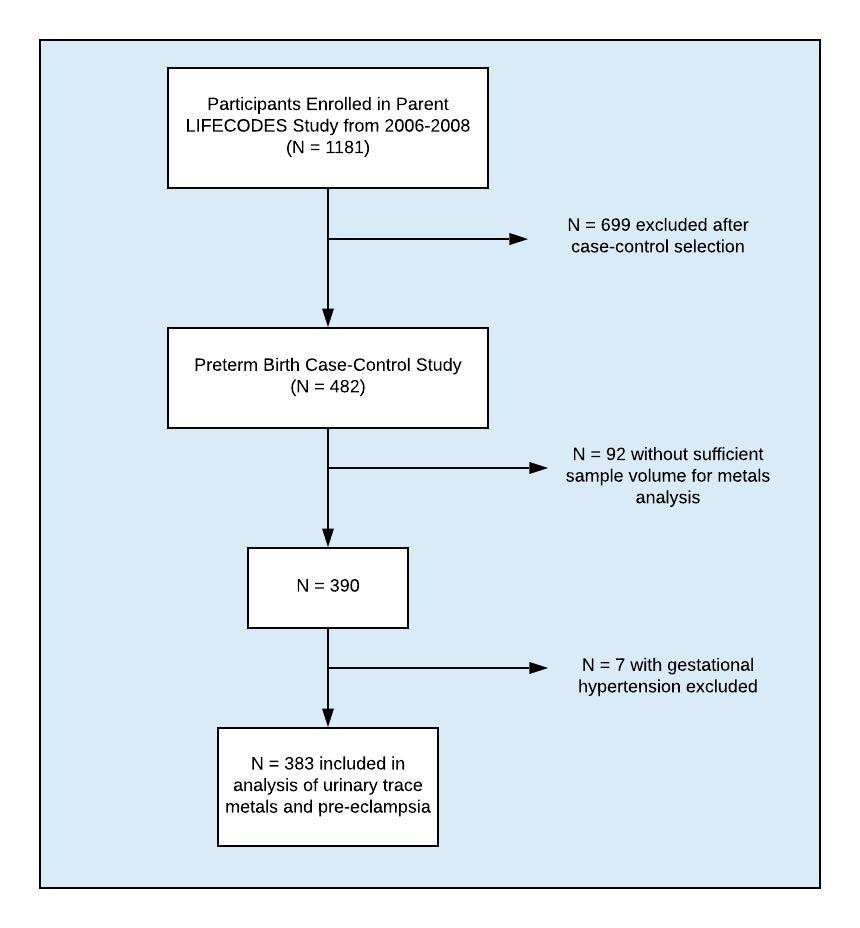
Supplemental Figure 1.** Flow-through diagram displaying study design.

**Supplemental Figure 2.** Simplified directed acyclic graph (DAG) used to identify potential confounders and/or colliders.
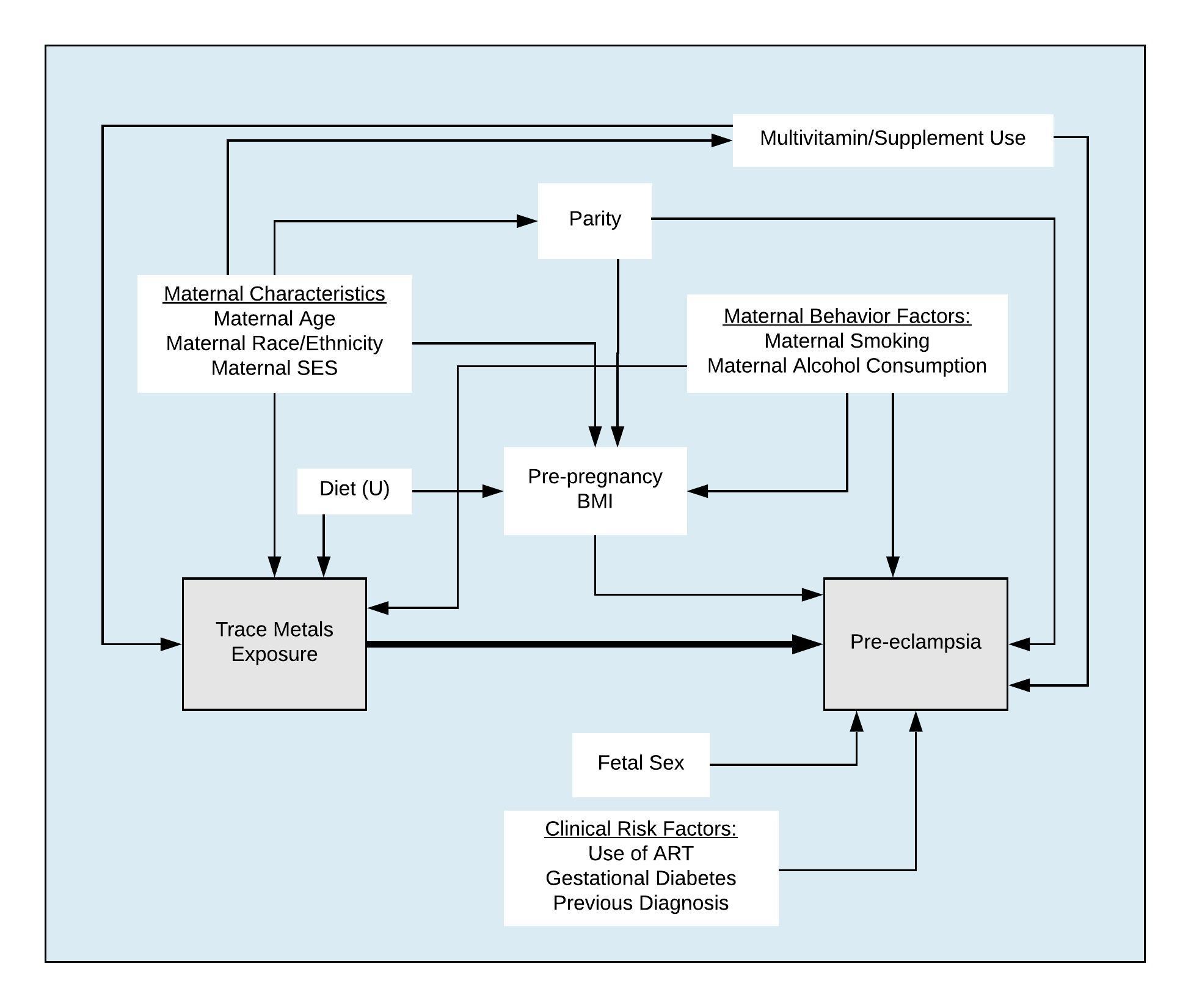


| **Supplemental Table 1.** Unadjusted and adjusted relationship between urinary metals and the HR (95% CI) of preeclampsia. | | | | | |
| --- | --- | --- | --- | --- | --- |
|  | **Unadjusted** | |  | **Adjusted^a^** | |
|  | HR (95% CI) | p |  | HR (95% CI) | p |
| *Single Contaminant Models* | |  |  |  |  |
| As | 0.70 (0.46, 1.09) | 0.11 |  | 0.72 (0.48, 1.08) | 0.11 |
| Ba | 1.09 (0.68, 1.74) | 0.72 |  | 0.98 (0.58, 1.65) | 0.94 |
| Cd | 0.93 (0.59, 1.46) | 0.75 |  | 0.91 (0.56, 1.48) | 0.69 |
| Cu | 1.15 (0.59, 2.23) | 0.67 |  | 0.88 (0.33, 2.40) | 0.81 |
| Hg | 0.91 (0.64, 1.30) | 0.61 |  | 0.83 (0.59, 1.18) | 0.31 |
| Mn | 1.07 (0.66, 1.74) | 0.79 |  | 1.15 (0.71, 1.87) | 0.56 |
| Mo | 0.60 (0.32, 1.15) | 0.12 |  | 0.56 (0.27, 1.13) | 0.10 |
| Ni | 0.90 (0.51, 1.60) | 0.72 |  | 0.79 (0.44, 1.44) | 0.45 |
| Pb | 1.02 (0.75, 1.40) | 0.89 |  | 0.91 (0.65, 1.28) | 0.59 |
| Se | 0.56 (0.18, 1.73) | 0.31 |  | 0.41 (0.13, 1.30) | 0.13 |
| Sn | 0.85 (0.56, 1.28) | 0.43 |  | 0.78 (0.47, 1.30) | 0.34 |
| Tl | 0.92 (0.57, 1.47) | 0.72 |  | 0.92 (0.55, 1.52) | 0.73 |
| Zn | 1.33 (0.74, 2.38) | 0.34 |  | 1.19 (0.61, 2.35) | 0.61 |
| Be^†^ | 0.96 (0.23, 3.99) | 0.95 |  | 0.72 (0.16, 3.22) | 0.67 |
| Cr^†^ | 1.83 (0.64, 5.22) | 0.26 |  | 2.83 (0.94, 8.48) | 0.06 |
| U^†^ | 1.34 (0.41, 4.38) | 0.63 |  | 0.74 (0.20, 2.79) | 0.65 |
| W^†^ | 0.79 (0.24, 2.55) | 0.69 |  | 0.90 (0.26, 3.07) | 0.87 |
| *Principal Components Analysis Models* | |  |  |  |  |
| PC1: Cu, Se, and Zn | 1.30 (0.68, 2.51) | 0.43 |  | 1.20 (0.53, 2.70) | 0.66 |
| PC2: Cd, Mn, and Pb | 1.42 (0.75, 2.71) | 0.28 |  | 1.39 (0.68, 2.87) | 0.37 |
| PC3: As, Hg, and Sn | 0.75 (0.41, 1.39) | 0.36 |  | 0.69 (0.36, 1.32) | 0.26 |

^*^Adjusted for specific gravity (for continuously measured metals in single contaminant models), smoking during pregnancy, race, educational attainment, insurance status, infant sex, ART, calcium supplementation, and gestational age at study visit.

^†^Denotes metals with >70% of samples below the limit of detection.

Abbreviations: HR, hazard ratio; CI, confidence interval; PC, principal component; As, arsenic; Ba, barium; Cd, cadmium; Cu, copper; Hg, mercury; Mn, manganese; Mo, molybdenum; Ni, nickel; Pb, lead; Se, selenium; Sn, tin; Tl, thallium; Zn, zinc; Be, beryllium; Cr, chromium; U, uranium; W, tungsten

| **Supplemental Table 2**. Unadjusted and adjusted^*^ relationship between urinary trace metals and the percent change (95% CI) in circulating maternal angiogenic biomarkers. | | | | | | | | | | | | | | | | | | | | | | | |  |  |
| --- | --- | --- | --- | --- | --- | --- | --- | --- | --- | --- | --- | --- | --- | --- | --- | --- | --- | --- | --- | --- | --- | --- | --- | --- | --- |
|  | | | | **Percent Change (95% CI)** | | | | | | | | | | | | | | | | | | | |  |  |
|  | **sFlt-1** | | | | | | | |  | | **PlGF** | | | | | | |  | **sFlt-1/PlGF Ratio** | | | | | |  |
|  | Unadjusted | | | | Adjusted | | | |  | | Unadjusted | | | Adjusted | | | |  | Unadjusted | | Adjusted | | | |  |
| *Single-Contaminant Model* | |  | | | | |  |  | | | |  | | | |  |  | | | | |  |  |  |  |
| As | -0.20 (-6.60, 6.64) | | | | -1.97 (-8.18, 4.66) | | | |  | | -0.08 (-6.85, 7.18) | | | -0.61 (-7.38, 6.66) | | | |  | -0.93 (-9.61, 8.59) | | -2.43 (-11.0, 7.01) | | | |  |
| Ba | -2.67 (-8.91, 3.99) | | | | -1.74 (-7.95, 4.88) | | | |  | | -3.59 (-10.1, 3.41) | | | -3.54 (-10.1, 3.50) | | | |  | 1.04 (-7.84, 10.8) | | 2.08 (-6.90, 11.9) | | | |  |
| Cd | -0.66 (-6.88, 5.98) | | | | -1.16 (-7.32, 5.40) | | | |  | | -6.87 (-13.0, -0.32) | | | -6.66 (-12.9, -0.01) | | | |  | 6.56 (-2.55, 16.5) | | 5.59 (-3.55, 15.6) | | | |  |
| Cu | 3.87 (-7.45, 16.6) | | | | 7.45 (-4.22, 20.6) | | | |  | | -18.8 (-27.7, -8.03) | | | -17.9 (-27.4, -7.21) | | | |  | 26.6 (8.05, 48.3) | | 30.1 (10.8, 52.7) | | | |  |
| Hg | 4.90 (-1.33, 11.5) | | | | 4.61 (-1.50, 11.1) | | | |  | | -3.22 (-9.30, 3.27) | | | -2.33 (-8.47, 4.21) | | | |  | 7.65 (-1.11, 17.2) | | 6.53 (-2.11, 15.9) | | | |  |
| Mn | 0.61 (-6.77, 8.58) | | | | -0.34 (-7.56, 7.45) | | | |  | | -2.22 (-9.80, 6.01) | | | -3.75 (-11.2, 4.37) | | | |  | 2.08 (-8.15, 13.5) | | 2.48 (-7.83, 13.9) | | | |  |
| Mo | -1.78 (-12.7, 10.5) | | | | -1.00 (-11.8, 11.1) | | | |  | | 6.94 (-5.59, 21.1) | | | 6.16 (-6.21, 20.2) | | | |  | -10.0 (-23.6, 5.94) | | -8.38 (-22.1, 7.77) | | | |  |
| Ni | -0.18 (-9.07, 9.58) | | | | 1.21 (-7.68, 11.0) | | | |  | | -1.32 (-10.6, 8.92) | | | -0.29 (-9.67, 10.1) | | | |  | 2.32 (-10.1, 16.4) | | 3.13 (-9.40, 17.4) | | | |  |
| Pb | -3.28 (-7.86, 1.51) | | | | -3.35 (-7.92, 1.44) | | | |  | | -7.62 (-12.2, -2.81) | | | -7.69 (-12.3, -2.81) | | | |  | 4.17 (-2.60, 11.4) | | 4.39 (-2.49, 11.8) | | | |  |
| Se | -10.4 (-26.1, 8.56) | | | | -14.2 (-29.0, 3.53) | | | |  | | -28.4 (-41.5, -12.5) | | | -29.1 (-42.0, -13.4) | | | |  | 24.8 (-4.34, 62.9) | | 20.4 (-7.70, 57.0) | | | |  |
| Sn | 5.59 (-0.77, 12.4) | | | | 3.83 (-2.58, 10.7) | | | |  | | 4.66 (-2.02, 11.8) | | | 4.01 (-2.89, 11.4) | | | |  | 0.92 (-7.43, 10.0) | | 0.02 (-8.59, 9.44) | | | |  |
| Tl | -5.48 (-12.3, 1.80) | | | | -5.43 (-12.0, 1.69) | | | |  | | -3.85 (-11.1, 4.03) | | | -4.66 (-11.8, 3.1) | | | |  | -2.11 (-11.7, 8.54) | | -0.82 (-10.5, 9.91) | | | |  |
| Zn | -7.01 (-15.0, 1.74) | | | | -6.68 (-14.9, 2.33) | | | |  | | -11.5 (-19.5, -2.68) | | | -9.82 (-18.3, -0.46) | | | |  | 5.40 (-6.99, 19.4) | | 4.59 (-8.17, 19.1) | | | |  |
| Be^†^ | -6.28 (-24.8, 16.9) | | | | -3.89 (-22.9, 19.8) | | | |  | | 21.3 (-3.90, 53.2) | | | 19.7 (-5.52, 51.8) | | | |  | -22.5 (-42.9, 5.15) | | -18.3 (-40.1, 11.4) | | | |  |
| Cr^†^ | -8.68 (-25.6, 12.1) | | | | -5.70 (-23.2, 15.8) | | | |  | | -24.3 (-38.7, -6.56) | | | -25.5 (-39.8, -7.74) | | | |  | 5.93 (-20.3, 40.7) | | 9.84 (-17.7, 46.7) | | | |  |
| U^†^ | -3.73 (-21.8, 18.5) | | | | -14.1 (-30.4, 6.01) | | | |  | | 1.56 (-18.5, 26.5) | | | -5.30 (-24.6, 18.9) | | | |  | -6.00 (-29.5, 25.4) | | -11.3 (-34.1, 19.5) | | | |  |
| W^†^ | 5.73 (-10.8, 25.3) | | | | 7.52 (-8.95, 27.0) | | | |  | | 4.55 (-12.7, 25.2) | | | 1.90 (-14.8, 21.9) | | | |  | 3.07 (-18.6, 30.4) | | 7.42 (-15.0, 35.8) | | | |  |
| *Principal Components Analysis Models* | | |  | | |  | | | |  | | |  | |  | | | | |  | | | | | |
| PC1: Cu, Se, and Zn | -0.35 (-6.85, 6.60) | | | | -0.03 (-6.62, 7.03) | | | |  | | -10.1 (-16.5, -3.30) | | | -10.6 (-17.0, -3.61) | | | |  | 11.3 (1.02, 22.6) | | 12.4 (1.78, 24.2) | | | |  |
| PC2: Cd, Mn, and Pb | 0.82 (-5.77, 7.88) | | | | 0.64 (-6.05, 7.81) | | | |  | | -6.94 (-13.5, 0.14) | | | -7.62 (-14.4, -0.34) | | | |  | 7.58 (-2.36, 18.5) | | 8.16 (-2.15, 19.5) | | | |  |
| PC3: As, Hg, and Sn | 4.71 (-1.79, 11.6) | | | | 2.51 (-4.00, 9.47) | | | |  | | 2.56 (-4.31, 9.93) | | | 1.15 (-5.91, 8.73) | | | |  | 1.45 (-7.46, 11.2) | | 0.47 (-8.69, 10.6) | | | |  |

^*^Adjusted for specific gravity (for continuously measured metals in single-contaminant models), smoking during pregnancy, race, educational attainment, insurance status, infant sex, ART, calcium supplementation, and gestational age at study visit.

^†^Denotes metals with >70% of samples below the limit of detection.

Abbreviations: sFlt-1, soluble fms-like tyrosine; PlGF, placental growth factor; HR, hazard ratio; CI, confidence interval; PC, principal component; As, arsenic; Ba, barium; Cd, cadmium; Cu, copper; Hg, mercury; Mn, manganese; Mo, molybdenum; Ni, nickel; Pb, lead; Se, selenium; Sn, tin; Tl, thallium; Zn, zinc; Be, beryllium; Cr, chromium; U, uranium; W, tungsten

| **Supplemental Table 3.** Standardized and rotated loading factors and communalities for each variable within each principal component. | | | | | |
| --- | --- | --- | --- | --- | --- |
|  | **Factor Loadings** | | | | |
|  | PC1 |  | PC2 |  | PC3 |
| As | 0.04 |  | 0.01 |  | **0.65** |
| Cd | 0.16 |  | **0.81** |  | 0.03 |
| Cu | **0.62** |  | 0.32 |  | 0.08 |
| Hg | -0.07 |  | 0.22 |  | **0.61** |
| Mn | -0.08 |  | **0.64** |  | 0.25 |
| Ni | 0.10 |  | 0.06 |  | -0.20 |
| Pb | 0.30 |  | **0.67** |  | -0.02 |
| Se | **0.78** |  | -0.09 |  | 0.15 |
| Sn | 0.22 |  | 0.01 |  | **0.66** |
| Tl | -0.03 |  | 0.13 |  | 0.19 |
| Zn | **0.75** |  | 0.19 |  | -0.02 |
| Eigenvalue | 1.73 |  | 1.73 |  | 1.41 |
| Total variance (%) | 22.6 |  | 11.9 |  | 11.5 |
| Cumulative (%) | 22.6 |  | 34.5 |  | 46.0 |
| Bold indicates a factor loading value > 0.40. | | | | | |
